# Geographic Inequalities in Accessing Improved Water and Sanitation Facilities in Nepal

**DOI:** 10.3390/ijerph16071269

**Published:** 2019-04-09

**Authors:** Chao Wang, Jing Pan, Sanni Yaya, Ram Bilash Yadav, Dechao Yao

**Affiliations:** 1 School of Public Policy & Management, China University of Mining and Technology, Xuzhou 221116, China; wangchaoccnu@163.com (C.W.); 11183895@cumt.edu.cn (J.P.); 2 Faculté de médecine, Université de Parakou, Parakou BP 123, Benin; Sanni.yaya@gmail.com; 3 Social Research Development Centre, Janakpur 03, Nepal; rambilashydv@gmail.com; 4 School of Public Administration, Hunan University of Finance and Economics, Changsha 410205, China

**Keywords:** water, sanitation, geographic inequality, Nepal, public health

## Abstract

In this study, we aimed to assess the geographic inequalities in access to improved water and sanitation facilities among Nepalese households. We conducted this study based on cross-sectional data obtained from Nepal Demographic and Health Surveys. The quality of water sources and sanitation were defined by World Health Organization (WHO) guidelines. The geographic categories used in the analyses included developmental region, ecological zone, and urbanicity. Percentages of households having access to improved toilet (5.6% in 1996 vs. 40.5% in 2016) and water (19.3% in 1996 vs. 27% in 2016) facilities has been increasing steadily since 1996 with a great proportion of the households still lacking access to these services. The number of households sharing the same toilet and traveling time to reach water sources have also decreased at the same time. Households in Far Western and Mountains had the lowest odds of having access to improved toilet and water facilities. Noticeable progress has been achieved in improving WASH (water, sanitation, and hygiene) coverage at national level, however, it is uneven across developmental and ecological zones. Households in the Far Western and Mountain regions appeared to be the most geographically disadvantaged in terms of having access to improved water and sanitation facilities.

## 1. Introduction

Sustainable provision of safe drinking water, sanitation, and hygiene (WASH) are the key perquisites for promotion of public health, quality of life, as well as strong indicators of human development standards [1,2,3]. One of the four targets of Millennium Development Goal (MDG) 7 was “To halve the proportion of the universal population without sustainable access to clean and safe drinking water and basic sanitation by 2015” [4]. While many countries were able to achieve appreciable progress through MDG and many other internationally propelled goals, a large number of countries are still lagging far behind in meeting their WASH targets. Notably among these countries are the ones in sub-Saharan Africa and South Asia. As one the poorest and most densely populated region on the globe, countries in South Asia are faced with looming water and sanitation crisis due to poor water supply and management infrastructure, industrial growth, uncontrolled urbanization, and environmental pollution. Contrary to expectations, Nepal is one of the most water insecure countries in the region despite being one of the richest in terms of per capita renewable water availability in Asia. Pollution of surface water resources through household and industrial refuge and open defecation are common scenarios in Nepal and across the South Asian region.

While perhaps less talked about, poor hygiene and sanitation are no less of the concern as water crisis for Nepal as the consequences of these are often closely interlinked and aggravative the impacts the others. Globally, unsafe hygiene and sanitation-related diseases account for 7% of total diseases burden and 19% of child mortality [2]. Regional statistics on disease burden attributable to hygiene and sanitation are not available for South Asian countries, however, there is substantial evidence of association between poor WASH and high rates of malnutrition and water-borne diseases (e.g., diarrhoea) among children [5,6,7,8]. A cross-sectional study in Nepal reported that about three-quarters of drinking water from school samples and two-fifths from community samples were contaminated with thermo-tolerant coliforms [9]. Poor knowledge and awareness of sanitation and routes of infection, as well as contamination of domestic water with human fecal organisms, are also common in the population [10,11]. Unhygienic living condition and usage of contaminated water for drinking and domestic usage have long-term negative impacts on nutrition and overall health status, especially for children [12,13]. The challenges of inadequate WASH capacity in Nepal is compounded by natural disasters such as arsenic contamination of ground water [14,15], earthquake [16,17], and recurrent flood. Loss of physical infrastructure and natural ecosystems due to natural disasters significantly heightens the vulnerability to and outbreak of WASH-related diseases.

Studies aimed at determining the predictors of access to WASH usually concentrate on the behavioral, political, socioeconomic, and development parameters. Despite a growing interest in the field of health geography that focuses on assessing the region-specific burden of a particular disease and the risk factors, little attention has been given to the regional disparities of WASH in South Asian studies. For countries as geographically diverse as Nepal, geographic position constitutes one of the most defining elements in access and utilisation of healthcare and other public amenities like safe water, sanitation, waste management. Although the government of Nepal has embarked on an ambitious plan of providing universal coverage of water and sanitation services for all by the end of 2017, making it a reality remains a far cry especially when the quality of the services is concerned.

Against the backdrop of the ongoing health policy and implementation efforts, persistently high rates of water-borne diseases and child mortality illustrate the need for continued investigation and monitoring the progress WASH coverage across the country. Nepal has a complex geophysical structure; hence, the nature and magnitude of the crisis are supposed to vary across regions. To date, there is little evidence on regional differences in access to improved water and sanitation facilities in Nepal. To this regard, we undertook the present study with an aim to describe the trend in household access to improved water and sanitation access at several levels including developmental region, ecological zone and urbanicity.

## 2. Methods

### 2.1. Study Setting

Nepal is a landlocked country in South Asia, ranking fifth in the region in terms of total population. The country boasts a rich terrestrial ecology and agricultural base, multiethnic culture, and a diverse geography with eight of the world’s ten tallest mountains. As a nation by the Himalayas, Nepal is blessed with great quantities renewable freshwater resources, and is a major source of annual flow of the Ganges. Despite the vast water resources and hydropower potential, the country is beset with serious water and energy insecurity and growing human development crisis. According to World Bank estimates, Nepal is one of the poorest countries in the world (GDP, 470 USD per capita), with more than two-fifths of the population living below the poverty line and about a quarter having access to sanitation [18]. Nepal consists of 75 districts and three ecological zones which are grouped under five development regions. Three ecological zones (Terai or lowlands, the Hills, and mountains) run parallel from east to west with significant variation in terms of demography, climactic pattern and biogeography of major habitats [19]. The development regions serve as administrative units to ensure proper allocation of resources and proportionate development across the regions.

### 2.2. Outcome and Explanatory Measures

Outcome variables were access to improved: (1) Water and (2) Sanitation facilities. We used World Health Organization (WHO) guidelines to classify the type of water and sanitation facilities as improved/unimproved (Table 1).

**Figure 1:**
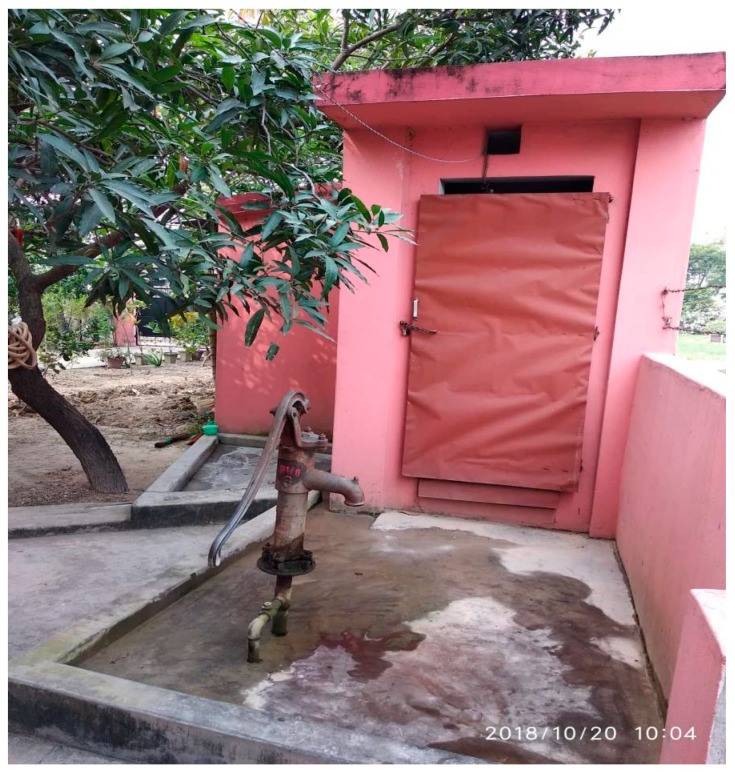
Backyard water source and toilet facility in a typical Nepalese household. Photo credit: Sudeep Sharma.

Figure 1 Shows the example of an improved source of water and toilet facility in a typical Nepalese household.

**Figure 2:**
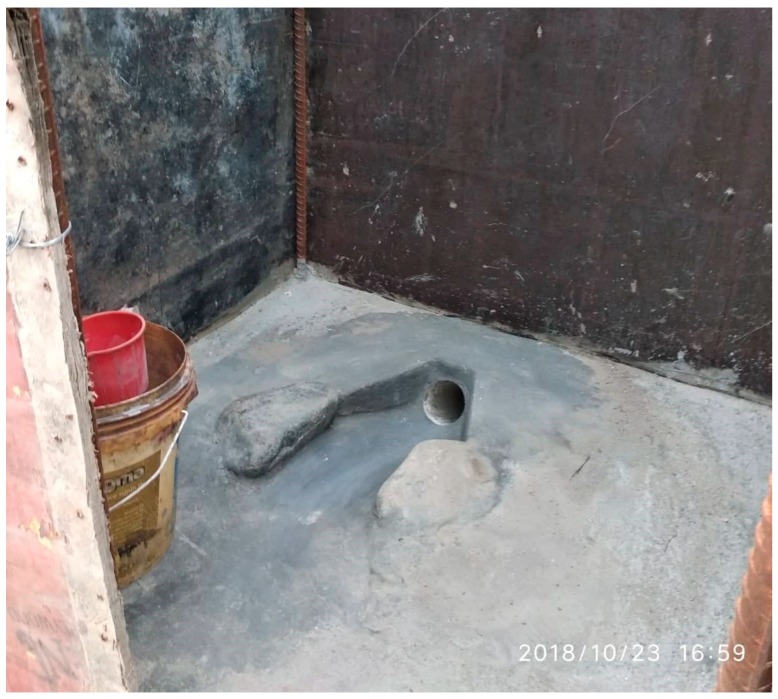
Example of a low-cost improved sanitation facility in a construction site. Photo credit: Sudeep Sharma.

Figure 2 shows the example of an improved and affordable toilet facility for temporary use in a construction site. 

Independent variables of interest were geographic inequality in access to water and sanitation. The datasets contained three different geographic/regional variables: (1) Developmental regions: Eastern, Central, Western, Midwestern, Far-western; (2) Ecological zone: Mountain, Hill, Terai; and (3) Urbanicity: Urban, rural.

Control variables: In order to measure independent associations between geographic variables and access to water and sanitation services, we considered several control variables to adjust the analysis for: Wealth index (Poorest, Poorer, Middle, Richer, Richest), Age (<30, 30–39, 40–49, 50–59, 59+), Sex (Male, Female), Education (No education, Primary, Secondary/higher).

### 2.3. Data Analysis

Data were analysed using SPSS version 24 (IBM, Armonk, NY, USA). At first, the datasets were checked for missing values and outliers and then merged to performed pooled analysis. Owing to the clustered structure of Demographic and Health Survey data, we used complex survey design method for all analyses. Sociodemographic variables were compared for both outcome variables (having access vs. not having access) by percentages with 95% CIs. Pearson correlations tests were used to measure statistical significance of these bivariate associations. The variables that showed significance at *p* ≤ 0.25 in the bivariate tests were retained for final regression analysis. The association between outcome (access to water and sanitation) and independent variables (developmental region, ecological zone, urbanicity) were measured by binary logistic regression models while controlling for the potentially confounding variables to produce the adjusted odds ratios. Results of regression analyses were presented as odds ratios with 95% CIs. All tests were two-tailed and associations were considered statistically significant at a *p*-value of <0.05.

### 2.4. Ethical Approval

All participants gave informed consent prior to taking part in the survey. Additional ethical approval was not necessary since the study was a secondary analysis of public domain data.

## 3. Results

### 3.1. Descriptive Statistics

The analysis included 47,257 households from five rounds of DHS across the country. Data on geographical location of the households and basic sociodemographic characteristics of the individual respondents were summarised in Table 2. Participation was highest from the Central region (30.5%) and lowest from Farwestern region (11%). Regarding the ecological zone, most of the interviewed households were from the Terai zone (43.3%), and a vast majority were located in the rural areas (68.8%). Regarding wealth status, 22.8% of the households were in lowest wealth quintile whereas 57.3% were in the non-poor category (middle, richer, and richest). Most of the respondents were aged between 30–39 years (24.6%), male (76.6%), had no formal education (47.8%). Respondents who reported having access to improved sanitation and water facilities were in the age groups of 30–39 years, male, and had Secondary/Higher level education (access to water was higher among those with no education).

Table 2 indicates that the overall percentages of households with access to improved sanitation and water facilities were respectively 47.7% (45.8–49.6) and 46.0% (43.7–48.3). The percentages were higher in the Western region, located in the Hill zone, and were rural residents. Percentage of both access to improve sanitation (5.6% in 1996 vs. 40.5% in 2016) and water has increased since 1996 (19.3% in 1996 vs. 27% in 2016).

**Figure 3:**
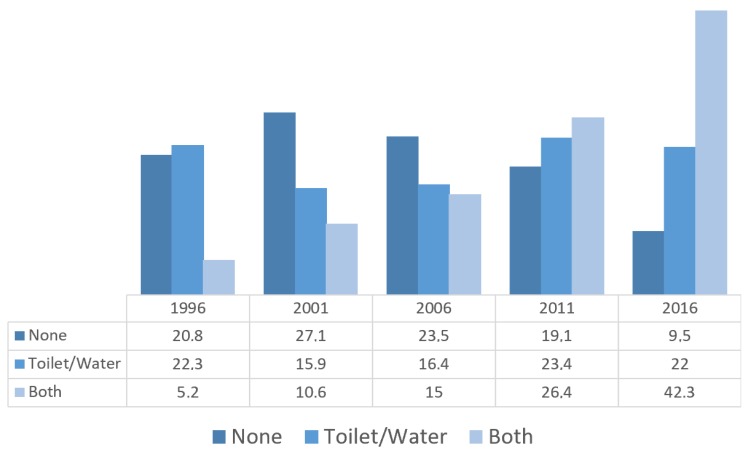
Progress towards coverage of improved water and sanitation access among Nepalese households 1996–2016.

Figure 3 illustrates that percentages of households lacking access to both water and sanitation facilities have more than halved since 1996 (20.8% in 1996 vs. 9.5% in 2016), while that of having access to both increased about eightfold during the same time (5.2% in 1996 vs. 42.3% in 2016).

**Figure 4:**
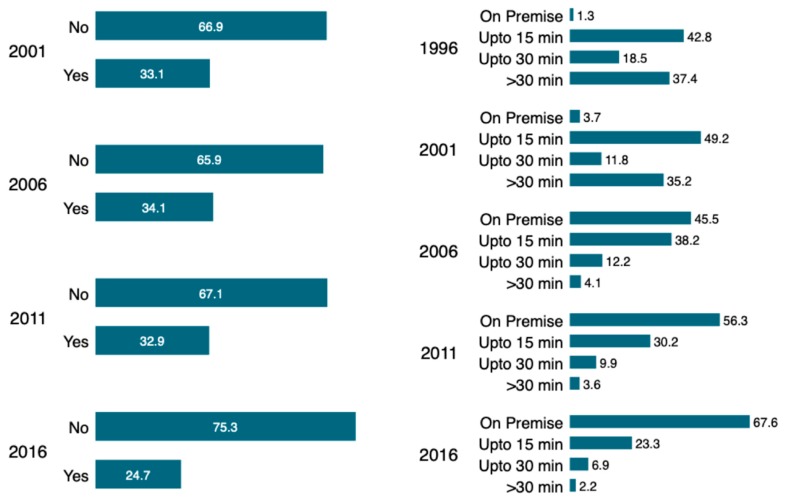
(**a**) Households sharing toilet facilities with other households and (**b**) time to reach water source.

Figure 4 depicts the trend in the proportion household sharing toilet facilities with others (a) and time to reach water source (b). It indicates that there is slow progress in the percentage of households sharing toilet facilities with others since 2006. However, these differences were significant at *p* < 0.05. There has been a substantial increase in the percentage of households having water facilities on premise. Nonetheless, a considerable proportion of the households still have to travel more than 15 min to reach sources of water.

**Figure 5:**
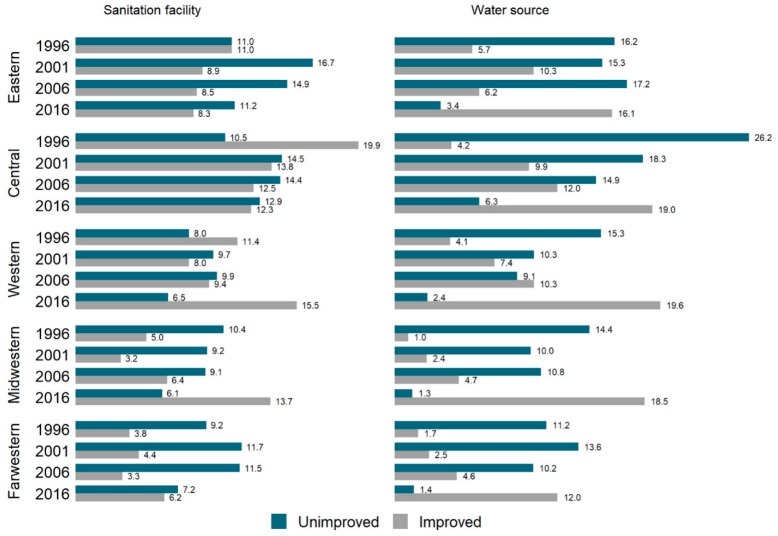
Trend in inequality in improved water and sanitation coverage across the developmental regions.

Figure 5 indicates that the proportional coverage of both toilet and water facilities have increased for Farwestern and Midwestern region since 1996, whereas that for Eastern and Central region have decreased considerably during the same time. (No data for Farwestern and Midwestern in 2011).

**Figure 6:**
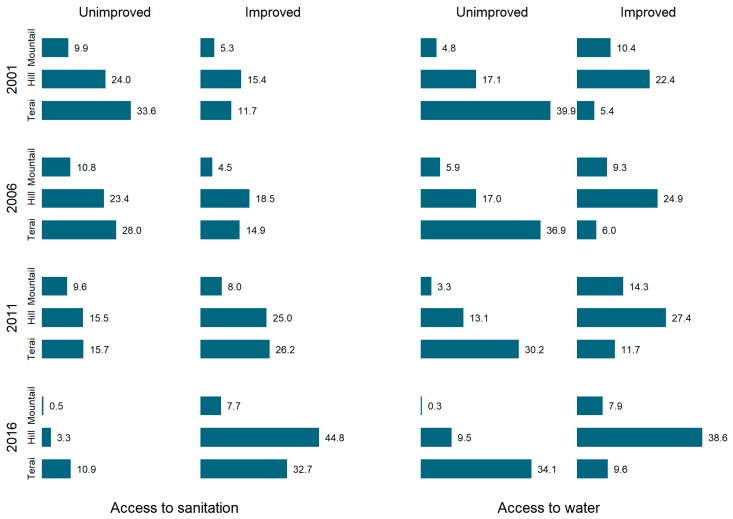
Trend in inequality in improved water and sanitation coverage across three ecological zones.

Figure 6 indicates a growing inequality in the coverage of sanitation and water across three ecological zones. There is a worsening trend for households in the mountain region while those in the Hills have experienced improvements since 1996. (Data were not available for 1996).

**Figure 7:**
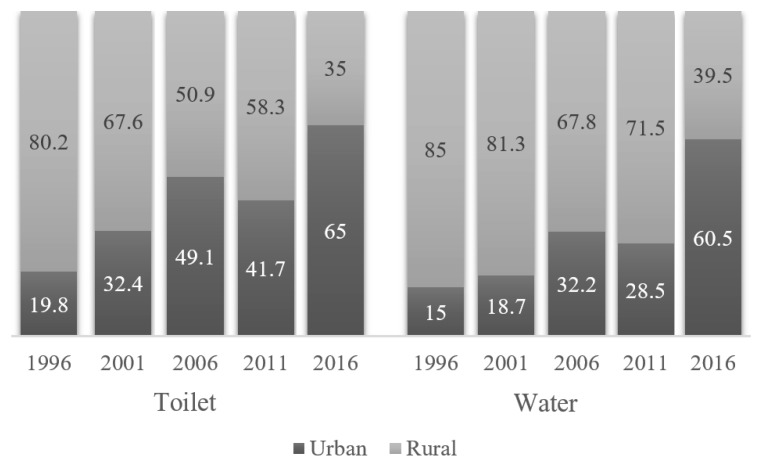
Trend in inequality in improved water and sanitation coverage between urban and rural areas.

As shown in Figure 7, the urban–rural gap in the coverage of sanitation and water has been decreasing since 1996.

**Figure 8:**
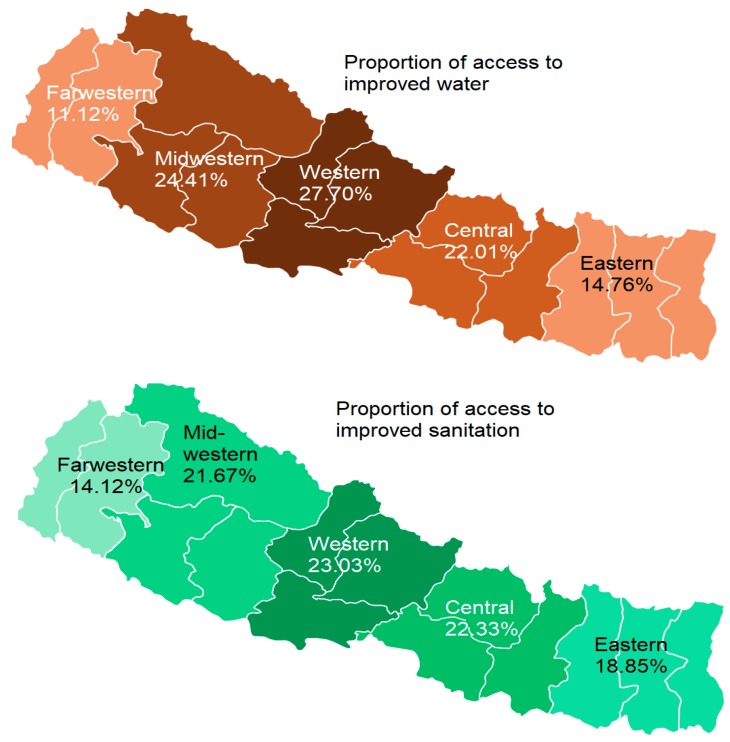
Over regional differences in improved sanitation and water access in 2016.

As shown in Figure 8, the proportion of households having access to improved sanitation was highest in the western region and lower in the peripheral regions, e.g., Farwestern (14.12%) and Eastern (18.85%). Similar to sanitation, the Western region also had the highest proportion of access to improved water (27.7%) and Farwestern had the lowest (11.12%).

### 3.2. Multivariable Regression Analysis

Results of regression analysis (Table 3) revealed significant geographical variation in lacking access to improved sanitation and water facilities. Compared with households in the Eastern region, those in the Western, Midwestern, and Far-western regions had higher odds of not having access to improved toilet and water facilities. Compared with households in the mountains, the odds of lacking access were significantly lower among those in the Terai zone (OR = 0.230, 95% CI = 0.115–0.446). Regarding urbanicity, the odds were significant for sanitation only; households in the rural areas were 26% less likely to lack access to toilet facilities (OR = 0.738; 95% CI = 0.679–0.801).

## 4. Discussion

Findings of the study provide evidence of significant regional and zonal disparities in the coverage of access to improve sanitation and water facilities among Nepalese households. In general, households located in the Eastern region, Terai ecological zone, and in rural areas were found to enjoy a wider coverage of WASH facilities compared with those in the other regions, whereas households in the Far West and mountain regions depict the worst scenario. Situated in the western-most end of the country, the Far West region is known for its difficult topography and is beset with host of development issues such as socioeconomic inequality, communal conflict, and poor public infrastructure [21,22]. Thus, it comes to no surprise that public health-related utilities such as municipal water infrastructure and services are also in meager supply around this corner of the country.

The percentage of households with access to both improved toilet and water facilities has increased steadily during last two decades, albeit at a slow pace, especially in the case of water. In 2016, slightly above two-fifths of the households had access to both, compared to about 5% back in 1996. Traveling time to get to water source has decreased substantially since 1996. Despite this progress, about a quarter of the households still had to travel more than 15 min to get water in 2016. Behind these achievements are the efforts by national (Department of Water Supply and Sewerage) as well as international development partners (e.g., UNICEF, World Bank, Plan International Nepal) who have been working towards community capacity building, promoting hygiene behaviour, disaster resilience, training of stakeholders, and technological and infrastructure development [23]. Overall, these findings suggest a slow and gradual improvement of the WASH situation and portray the tenacious nature of water and sanitation poverty in the country.

Previous studies have reported regional inequality in access to WASH for sub-Saharan countries [24,25]. However, similar evidence is not available for Nepal. One possible reason behind this could be absence of quality data, and funding and research constraints. In this study, we made use of country-representative datasets containing general information on household and living conditions which can serve as an important resource for monitoring national progress in WASH. The data were secondary, and therefore it is possible that some contextual information was not collected that could help understand the causes that underlie these variations. So, it is recommended that some of the findings are interpretation with caution. Contrary to expectation, we found that educational status of the respondents did not show any protective effect in having access to improved water facilities. Even more surprisingly, rural households were found to have higher prevalence of access to both improved water and toilet facilities. The reasons behind this are not possible to deduce from the present analysis, and could be indicative of high rates of urban poverty and outcome of rapid and unplanned urbanisation. While an increasing number of people are being pulled by the glamour of urban life and choosing service sector jobs, city planners are struggling to create housing, transportation, and sanitation infrastructure for the incoming urban dwellers.

Although our data were cross-sectional, and the findings are temporal, they bear important policy implications for WASH programs, researchers, and policy makers. Understanding regional inequalities help identify the gaps and limitations in resource allocation and program implementation. Being a country characterised by high rates of poverty, widespread child malnutrition, and frequent natural disaster, investing in WASH needs to be regarded as an urgent health and development imperative. Results of our study underscore the need for keeping in mind the geography of water and sanitation poverty. Inequities in sanitation coverage translate into health inequities across socio-economic groups [26]. Investing in developing WASH infrastructure should be regarded as a priority agenda as it is hailed as an important intervention strategy for reducing morbidity, mortality, and health care expenditure [2].

Water crisis in Nepal is a rather complex issue due to poor infrastructure and management, and the lack of institutions for providing technical support in crisis situations. Policy makers should work towards enhancing the capabilities to ensure sustainable provision of and access to improved water supplies. Numerous national and international organisations are contributing to water and sanitation infrastructure development and freshwater resource management strategies. Ensuring and reinforcing political transparency will be required to make sustainable use of available water resources and prevent further degradation of water ecosystems. Resolving any existing water conflict with neighbouring countries is also important.

### Strengths and Limitations

As far as we are concerned, this is the first study to report geographic inequality in the coverage of improved water and sanitation on a nationally representative sample in Nepal. These findings on regional variation in WASH coverage can be instrumental for ongoing and future projects to ensure the equitable provision of sustainable sanitation and hygiene services across the country. The data were cross-sectional, but were large enough to help make meaningful conclusions, and more so since the samples were selected nationwide. One particular strength is that that analysis was adjusted for several important confounders including household wealth status and educational status of the respondents. However, as the data were secondary, we did not have control over the selection of variables. There were no indicators of personal hygiene (e.g., handwashing) and hygiene-related behaviour among respondents. More studies should be performed to explore the geopolitical factors that may underlie the regional disparities.

## 5. Conclusions

The coverage of improved sanitation and water facilities has improved considerably during past two decades; however, there is still a long way to go as a large proportion of the households still living without sustainable access to safe drinking water and toilet facilities. Findings also indicate growing regional disparities in accessing sanitation and water facilities. While the urban–rural gaps have been decreasing, that among the developmental region and ecological zones are rising. Based on these observations, it is suggestible that considering WASH as an integral component of health and public policies can facilitate the achievement of universal coverage of these services. Stronger political commitment and transparency in program implementation is crucial to fighting the persistent water and sanitation poverty in the population. More studies should be carried out to explore the situation in the slum areas, and to estimate the impacts of natural disasters and climate change on sanitation and water security in the country.

## Figures and Tables

**Table 1:** World Health Organization (WHO) classification of improved sanitation and water supply.

Type of Facility	Unimproved	Improved
Sanitation	Unimproved sanitation facilities: do not ensure hygienic separation of human excreta from human contact. Unimproved facilities include pit latrines without a slab or platform, hanging latrines and bucket latrines.	Improved sanitation facilities: ensure hygienic separation of human excreta from human contact. They are use of the following facilities: Flush/pour flush to: piped sewer system, septic tank, pit latrine; Ventilated improved pit (VIP) latrine, Pit latrine with slab, Composting toilet.
Water	unimproved drinking-water sources: Unprotected dug well, unprotected spring, cart with small tank/drum, surface water (river, dam, lake, pond, stream, canal, irrigation channels), and bottled water.	Other improved drinking-water sources: Public taps or standpipes, tube wells or boreholes, protected dug wells, protected springs or rainwater collection. Piped water on premises: Piped household water connection located inside the user’s dwelling, plot or yard.

Source: WHO/UNICEF Joint Monitoring Programme for Water Supply and Sanitation, 2010. ISBN 978 92 4 156395 6 (NLM classification: WA 670) [20].

**Table 2:** Geographic and sociodemographic profile of the households and respondents.

Variables	Definitions	(*N* = 47,257) (%)	Access to Improved Sanitation 47.7% (95% CI = 45.8–49.6)	Access to Improved Water 46.0% (95% CI = 43.7–48.3)
Year				
1996	Year of conducting the field work	8082, 17.1	5.6 (4.7, 6.6)	19.3 (17.7, 20.9)
2001		8602, 18.2	11.6 (10.5, 12.9)	14.0 (12.4, 15.8)
2006		8707, 18.4	14.9 (12.9, 17.1)	16.4 (14.1, 19.0)
2011		10,826, 22.9	27.4 (24.4, 30.6)	23.3 (20.6, 26.3)
2016		11,040, 23.4	40.5 (37.4, 43.7)	27.0 (24.3, 29.9)
*p*-value			<0.001	<0.001
Developmental region				
Eastern	Divisions based on administrative goals and state of socioeconomic development	10,063, 21.3	18.6 (16.4, 21.0)	17.9 (15.7, 20.3)
Central		14,412, 30.5	36.8 (33.8, 40.0)	42.7 (39.6, 45.8)
Western		11,731, 24.8	31.0 (28.1, 34.0)	25.3 (22.5, 28.2)
Midwestern		5847, 12.4	8.1 (6.7, 9.7)	9.2 (7.6, 11.1)
Far-western		5204, 11	5.5 (4.7, 6.3)	4.9 (4.2, 5.8)
*p*-value			<0.001	<0.001
Ecological zone *				
Mountain	Divisions based on biogeographical and climactic patterns	5450, 13.9	6.8 (5.5, 8.3)	12.6 (10.8, 14.7)
Hill		16,748, 42.8	52.1 (48.6, 55.5)	68.5 (64.8, 71.9)
Terai		16,977, 43.3	41.2 (37.8, 44.6)	18.9 (15.8, 22.4)
*p*-value			<0.001	<0.001
Urbanicity				
Urban	Whether the household if located in rural or urban site	14,721, 31.2	41.5 (38.7, 44.4)	38.4 (35.5, 41.5)
Rural		32,536, 68.8	58.5 (55.6, 61.3)	61.6 (58.5, 74.5)
*p*-value			0.230	<0.001
Wealth index *				
Poorest	Index of relative wealth status of households based on the possession of durable goods (e.g., refrigerator and TV) and building material (e.g., concrete and wooden), rather than personal income	6970, 22.8	11.6 (10.1, 13.3)	23.5 (21.1, 26.0)
Poorer		6070, 19.9	13.2 (12.0, 14.6)	19.6 (18.0, 21.4)
Middle		5381, 17.6	15.4 (14.2, 16.7)	15.0 (13.5, 16.6)
Richer		5822, 19	25.2 (23.5, 27.0)	17.4 (15.5, 19.5)
Richest		6330, 20.7	34.6 (32.0, 37.4)	24.5 (21.5, 27.8)
*p*-value			<0.001	<0.001
Age				
<30	Respondent’s age in completed year at the time of the survey	7912, 16.7	16.1 (15.2, 17.0)	17.1 (16.2, 17.9)
30–39		11,606, 24.6	23.7 (23.0, 24.5)	23.4 (22.7, 24.1)
40–49		10,478, 22.2	21.9 (21.3, 22.6)	22.0 (21.4, 22.6)
50–59		8634, 18.3	18.9 (18.2, 19.6)	18.3 (17.7, 18.9)
59+		8627, 18.3	19.3 (18.6, 20.1)	19.2 (18.5, 20.0)
*p*-value			<0.001	<0.001
Sex				
Male	Sex of the respondent	36,217, 76.6	73.4 (72.4, 74.3)	74.9 (74.0, 75.8)
Female		11,040, 23.4	26.6 (25.7, 27.6)	25.1 (24.2, 26.0)
*p*-value			<0.001	<0.001
Education				
No education	Highest educational level obtained by the respondents categorised in terms of number of schooling years	22,590, 47.8	34.3 (33.1, 35.6)	44.1 (42.7, 45.5)
Primary		10,468, 22.1	21.8 (21.0, 22.6)	23.0 (22.3, 23.8)
Secondary/higher		10,500, 22.2	43.9 (42.4, 45.3)	32.9 (31.4, 34.4)
*p*-value			<0.001	<0.001

N.B. * = Numbers do not add up due to missing observations. *p*-values are from Pearson’s Chi-Square tests.

**Table 3:** Predictors of lacking access to improved sanitation and water in Nepal.

Region	Access to Improved Sanitation				Access to Improved Water			
	Sig.	OR	95% CI		Sig.	OR	95% CI	
			Lower	Upper			Lower	Upper
Region (Eastern)								
Central	0.559	0.970	0.874	1.075	0.077	0.923	0.845	1.009
Western	<0.001	2.046	1.827	2.292	<0.001	1.780	1.623	1.953
Midwestern	<0.001	3.346	2.920	3.833	<0.001	1.364	1.224	1.520
Far-western	<0.001	3.062	2.655	3.532	<0.001	0.600	0.535	0.673
Ecological zone (Mountain)								
Hill	0.860	0.990	0.886	1.106	<0.001	0.457	0.415	0.503
Terai	<0.001	0.230	0.115	0.446	<0.001	0.234	0.131	0.337
Urbanicity (Urban)								
Rural	<0.001	0.738	0.679	0.801	0.116	0.948	0.887	1.013
Nagelkerke R^2^	0.618				0.575			

N.B. Regression models are adjusted for the sociodemographic and household factors.
